# Immune-Related lncRNAs Pairs to Construct a Novel Signature for Predicting Prognosis in Gastric Cancer

**DOI:** 10.3389/fsurg.2022.807778

**Published:** 2022-03-23

**Authors:** Tianshang Bao, Zeyu Wang, Jia Xu

**Affiliations:** Department of Gastrointestinal Surgery, Renji Hospital, School of Medicine, Shanghai Jiao Tong University, Shanghai, China

**Keywords:** checkpoint blockade therapy, gastric cancer (GC), long non-coding RNA, TCGA, tumor-infiltrating immune cell

## Abstract

**Background:**

Immune-related long non-coding RNAs (irlncRNAs) appear valuable in predicting prognosis in patients with cancer. In this study, we used a fresh modeling algorithm to construct irlncRNAs signature and then assessed its predictive value for prognosis, tumor immune infiltration, and chemotherapy efficacy in gastric cancer (GC) patients.

**Materials and Methods:**

The raw transcriptome data were extracted from the Cancer Genome Atlas (TCGA). Patients were randomly divided into the training and testing cohort. irlncRNAs were identified through co-expression analysis, after which differentially expressed irlncRNA (DEirlncRNA) pairs were identified. Next, we developed a model to distinguish between high- or low-risk groups in GC patients through univariate and LASSO regression analyses. A ROC curve was used to verify this model. After subgrouping patients according to the median risk score, we investigated the connection between the risk score of GC and clinicopathological characteristics. Functional enrichment analysis was also performed.

**Results:**

We find that the results indicate that immune-related lncRNA signaling has essential value in predicting prognosis, and it may be potential to measure the Efficacy for immunotherapy. This feature may be a guide to the selection of GC immunotherapy.

**Conclusion:**

Our data revealed that immune-related lncRNA signaling had essential value in predicting prognosis, and it may be potentially used to measure the efficacy for immunotherapy. This feature may also be used to guide the selection of GC immunotherapy.

## Introduction

Gastric cancer (GC) is the 5th most common neoplasm and the 3rd most deadly cancer worldwide (1–6). Although the relative 5-year survival rate for GC has significantly improved over the years, the prognosis of GC patients remains low, with the overall 5-year relative survival rate being approximately 20%. The major risk factors for GC include *Helicobacter pylori* infection, age, high salt intake, and low intake of fruits and vegetables (7, 8). At present, surgery is considered the mainstream treatment for primary gastric carcinoma. Yet, as there is a low rate of early diagnosis, most patients tend to miss the optimal surgical window. Radiotherapy and chemotherapy are commonly used for advanced-stage cancer. Nevertheless, these types of treatments have been associated with certain side effects. Therefore, identifying new biomarkers is crucial to enhance early diagnosis and increase prognosis and treatment.

Over the years, immunotherapy has been widely applied to treat patients with advanced gastric cancer. New strategies have been based on targeting or manipulating the immune system to reactivate the function of anti-tumor (9, 10). Considerable advances have been made in the pharmacological treatment of GC, particularly with immune checkpoint inhibitors (ICIs) (11). One of the most important breakthroughs was achieved using humanized monoclonal antibodies against programmed cell death-1/programmed cell death-ligand 1 (PD-1/PD-L1) and cytotoxic T-lymphocyte–associated antigen 4 (CTLA-4), such as ipilimumab (anti-CTLA-4), nivolumab, pembrolizumab (anti-PD-1), and avelumab, durvalumab, atezolizumab (anti-PD-L1) (10, 12, 13), which reverse T cell exhaustion and represent a powerful anti-tumor immune response (14).

Human transcriptome includes many non-coding RNAs, including long non-coding RNAs (lncRNAs) with a length of more than 200 nucleotides (15). These small RNAs lack an open reading framework and cannot encode proteins. lncRNAs regulate gene expression at the transcriptional, post-transcriptional, and epigenetic levels. Their function is directly related to cell localization; they interact with DNA, RNA, and protein (16, 17). These interactions affect many cellular processes, including cell growth and development, and promote the proliferation of cancer cells (18, 19). With the emergence of new sequencing technologies, increasing data have revealed that lncRNAs exert a novel role in tumor biology (20). As a new prognostic and diagnostic biomarker, lncRNA has excellent clinical application prospects. Given that the number of non-coding RNAs far exceed protein-coding genes and show a high degree of tissue and cancer type specificity, characterizing new lncRNA targets may revolutionize cancer treatment. In addition, recent evidence indicated that lncRNAs influence the malignant phenotype of cancer through alterations in the genome or transcriptome and changes in the immune microenvironment.

The accuracy of prognostic cancer models based on the combination of two biomarkers is superior to a single gene marker (21). In this study, we used a fresh modeling algorithm to construct irlncRNAs signature and then assessed its predictive value in prognosis, tumor immune infiltration, and chemotherapy efficacy in gastric cancer (GC) patients.

## Materials and Methods

### Data Collection

The data of RNA expression profiles and clinical features for GC were downloaded from The Cancer Genome Atlas (TCGA) program (https://portal.gdc.cancer.gov/repository), including 375 GC tissues and 32 non-tumor tissues. Immune-related genes (ir-genes) were obtained from the ImmPort database (http://www.immport.org). A co-expression strategy was used to identify irlncRNAs. The immune gene correlation coefficients above 0.4 and *P*-values < 0.001 were considered to be irlncRNAs.

Using the R package *caret*, 375 gastric cancer patients were randomly distributed into a training group (*n* = 225) and a test group (*n* = 150). Differential expression analysis between irlncRNAs performed using the R package *Dseq2* identified the differentially expressed irlncRNA (DEirlncRNA) in the training cohort with log fold change |FC| > 2.0 and *p*-value < 0.05.

### Pairing Differentially Expressed irlncRNA (DEirlncRNA)

A 0-or-1 matrix was constructed by cyclically singly pairing the DEirlncRNA. The matrix was defined as 1 if the previous lncRNA had a higher expression than the latter; otherwise, it was defined as 0. A DEirlncRNA pair was considered as a valid pair when the number of pairs whose expression was set to 0 or 1 exceeded 20% of the overall number or was less than 80%.

### Establishment of a Risk Model and Calculation of Risk Score

Univariate Cox analysis was performed to evaluate the link between DEirlncRNA pairs and overall survival (OS) of GC samples in the training group. Eighty-one of DEirlncRNA pairs were significantly associated with OS. Through least absolute shrinkage and selection operator (LASSO) regression analysis (via *glmnet* in the R software), 31 DEirlncRNA pairs showed high significance. Finally, 10 DEirlncRNA pairs were selected, and multivariate Cox regression analysis was used to construct a prognostic model. The following formula was used: risk score = (0.42791350 ^*^ RP11-613D13.8|RP11-576I22.2 expression) + (0.91712368 ^*^ HOXA11-AS|CDIPT-AS1 expression) +(0.44998528 ^*^ MIR663AHG|CDIPT-AS1 expression) + (−0.37327021 ^*^ PART1|HAND2-AS1 expression) + (0.62534486 ^*^ AP000695.6|RP11-492E3.2 expression) + (−0.81336299 ^*^ LA16c-325D7.1|RP11-884K10.6 expression) +(−0.76594801 ^*^ HOXC-AS1|LINC00460 expression) + (−0.24445084 ^*^ RP4-760C5.3|MIR663AHG expression) + (−0.47892575^*^ RP11-1069G10.1|FLG-AS1 expression) + (−0.18018873 ^*^ CTD-2529O21.1|BVES-AS1 expression).

In all cohorts, patients were grouped into low and high-risk groups according to the median risk score. Modeled area under curves (AUCs) and receiver operating characteristic (ROC) curves were plotted at 1, 2, and 3 years using the “*survivalROC*” package. Kaplan-Meier method was used to assess survival differences between the high and low-risk groups.

### Validity of the Constructed Risk Model

In order to prove feasibility of randomly grouping stomach adenocarcinoma (STAD) patients from TCGA as the validation group, 10-fold cross validation was used to test algorithm accuracy of the prognosis model. Divide STAD dataset into ten parts, nine of which were used as training data and one as testing data in turn. The corresponding average AUC was obtained. The R packages used in these steps were *caret* and *proc* package.

Differential expression analysis between irlncRNAs performed using the R package *Dseq2* identified the differentially expressed irlncRNA (DEirlncRNA) in the training cohort with log fold change |FC| > 2.0 and *p*-value < 0.05.

The association between risk models and clinicopathological characteristics was analyzed using Chi-square tests. The Wilcoxon signed-rank test was used to assess differences in risk scores between groups for these clinicopathological characteristics. Univariate Cox regression analyses of risk scores and clinicopathological characteristics for the three cohorts were then used to verify whether the model could be used as an independent predictive factor for clinical prognosis. The results were demonstrated in a forest plot. Finally, we also performed nomograms based on multivariate regression analysis via the *rms* package in R software and drew line segments with the scale on the same plane according to proportion for visualization. The R packages used in these steps were *pHeatmap survival, rms*, and *ggupbr*.

### Investigation of Tumor-Infiltrating Immune Cells

Different methods, including CIBERSORT, MCPCOUNTER, XCELL, QUANTISEQ, TIMER, CIBERSORT-ABS, and EPIC were used to calculate the immune infiltration status of samples in the STAD dataset (TCGA) and explore the association between immune cell characteristics and risk. Wilcoxon signed-rank test was used to assess immune infiltrating cell types between the low and high-risk groups. Spearman correlation analysis was used to explore the association between immune infiltrating cells and risk score values. The threshold of significance was set at *P* < 0.05. The R *ggplot2* software package was utilized in this procedure.

### Exploration of the Implications of the Model in the Clinical Treatment

To assess the model in the clinical treatment of gastric cancer, we measured the IC_50_ of common administrating chemotherapeutic drugs in the STAD dataset (TCGA). The American Joint Committee on Cancer (AJCC) guidelines recommend chemotherapeutic drugs such as cisplatin, docetaxel, paclitaxel, mitomycin, and doxorubicin for GC treatment. Through the calculation of the Wilcoxon signed-rank test, the discrepancy in the IC_50_ between the high-risk and low-risk groups was obtained. By using “*pRRophetic*” and “*ggplot2*” of R package, the results were shown in the form of a box plot.

### Characterization of the Expression of Immunosuppressive Molecules in the Context of ICIs

*The ggstatsplot*′ R package was used to analyze the association between the expression level of genes related to ICIs and the novel model, and the results were visualized in the format of a violin plot.

### Functional Enrichment Analysis

Kyoto Encyclopedia of Genes and Genomes (KEGG) and Gene Set Enrichment Analysis (GESA) pathway enrichment analyses were performed in R using the function of *clusterProfiler*. The threshold of significance was set at *p* < 0.05.

## Results

### Identification of DEirlncRNAs

**Figure 1:**
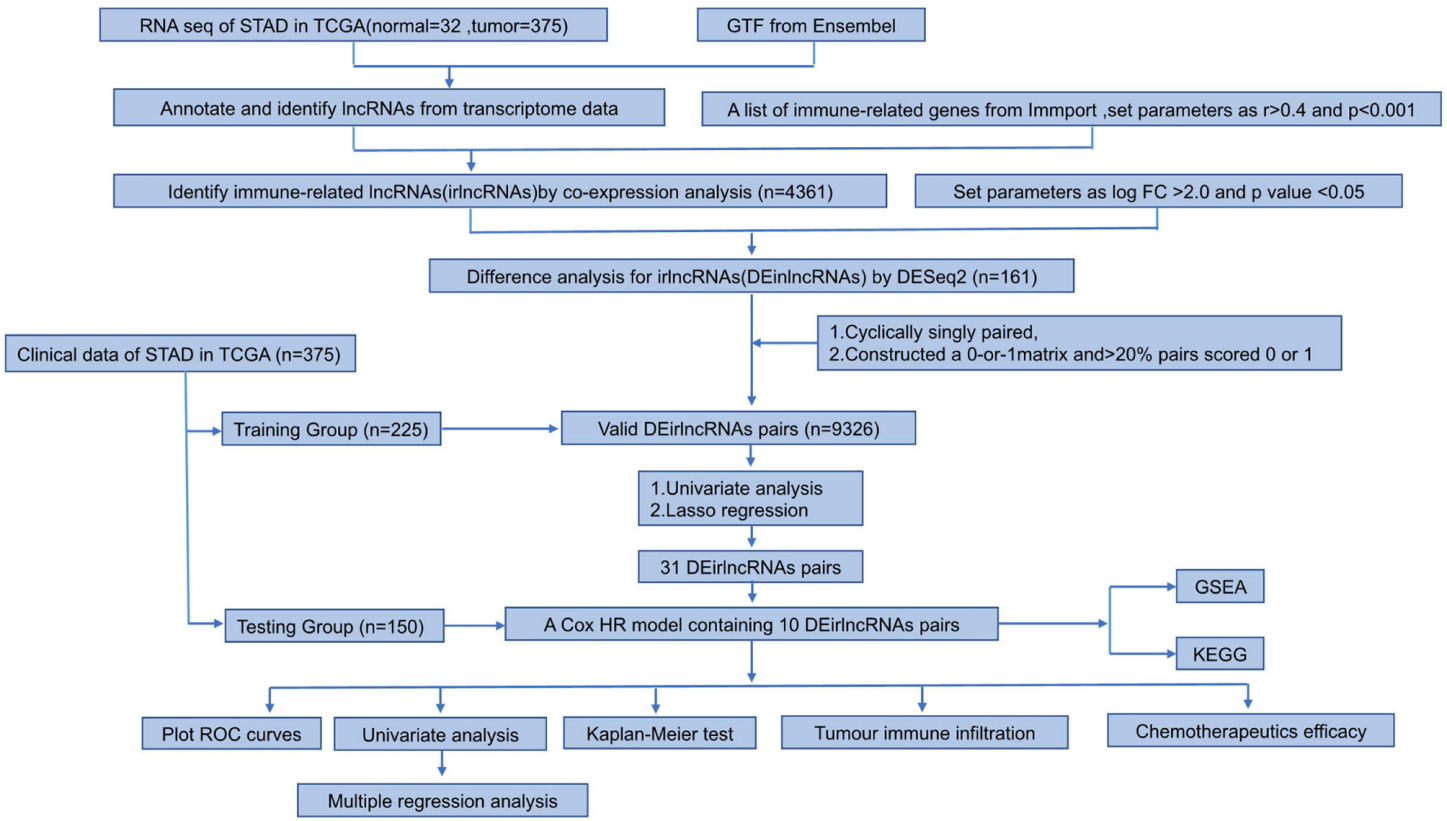
Flow chart of this study.

The study process is shown in Figure 1. The raw transcriptome data from 375 GC and 32 normal samples were extracted from The Cancer Genome Atlas (TCGA). The clinicopathological data of 375 GC patients, including sex, age, pathological data (tumor staging, grading, and TNM staging), survival time, and survival status, were also obtained (Table 1).

**Table 1:** Clinicopathological characteristics in patients with GC from TCGA (*n* = 375).

**Variables**	**Subtype**	**Patients (%)**
**Age (years)**	≥68	194 (51.7%)
	<68	173 (46.1%)
	Unknow	8 (2.2%)
**Gender**	Male	241 (64.3%)
	Female	134 (35.7%)
**Stage**	I	53 (14.1%)
	II	111 (29.6%)
	III	150 (40.0%)
	IV	38 (10.1%)
	Unknow	23 (6.1)
**Grade**	G1	10 (2.7%)
	G2	137 (36.5%)
	G3	219 (58.4%)
	Unknow	9 (2.4%)
**T**	T1	18 (4.8%)
	T2	74 (19.7%)
	T3	162 (43.2%)
	T4	97 (25.9%)
	Unknow	24 (6.4%)
**N**	N0	111 (29.6%)
	N1	89 (23.7%)
	N2	73 (19.5%)
	N3	73 (19.5)
	Unknow	29 (7.7%)
**M**	M0	311 (82.9%)
	M1	24 (6.4%)
	Unknow	40 (10.7%)
**Survival status**	Alive	300 (80.0%)
	Dead	75 (20.0%)

**Figure 2:**
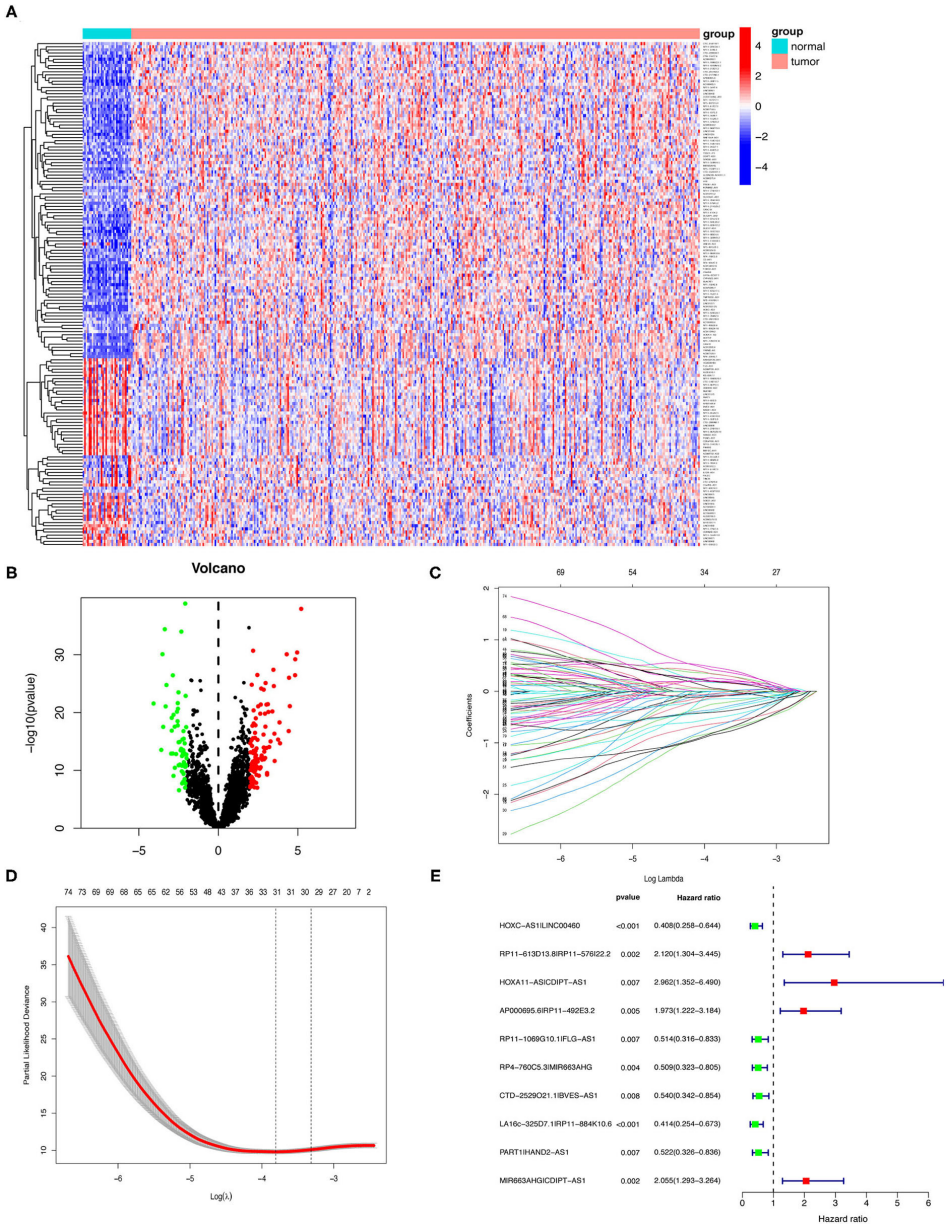
Establishment of a risk assessment model using DEirlncRNA pairs. **(A,B)** The heatmap **(A)** and a volcano plot **(B)** showed 161 DEirlncRNA in normal and GC tissues from the TCGA database. The colors from blue to red in the heat map represent low to high expression levels. Red and green dots represent up- and down-regulated irlncRNA, respectively. Black dots indicate no differential expression of irlncRNA. **(C)** LASSO Cox regression model profiles of 81 DEirlncRNA pairs selected by univariate Cox regression analysis in the training group. **(D)** The penalty coefficient was utilized to minimize the mean square error of the model. **(E)** A forest plot of the 10 DEirlncRNA pairs determined through multivariable Cox regression analysis. Unadjusted HRs are shown with 95% confidence intervals.

Next, we annotated the data by gene transfer format (GTF) files from Ensembel. We performed differential expression analysis of lncRNAs between cancer tissues and normal tissues. Among them, 4,361 irlncRNAs were obtained (Supplementary Table 1) by the co-expression analysis of lncRNA and ir-genes. Finally, 161 DEirlncRNAs (102 upregulated and 59 downregulated DEirlncRNAs) were selected (Figures 2A,B).

### Establishment of DEirlncRNA Pairs and a Risk-Assessed Model

**Figure 3:**
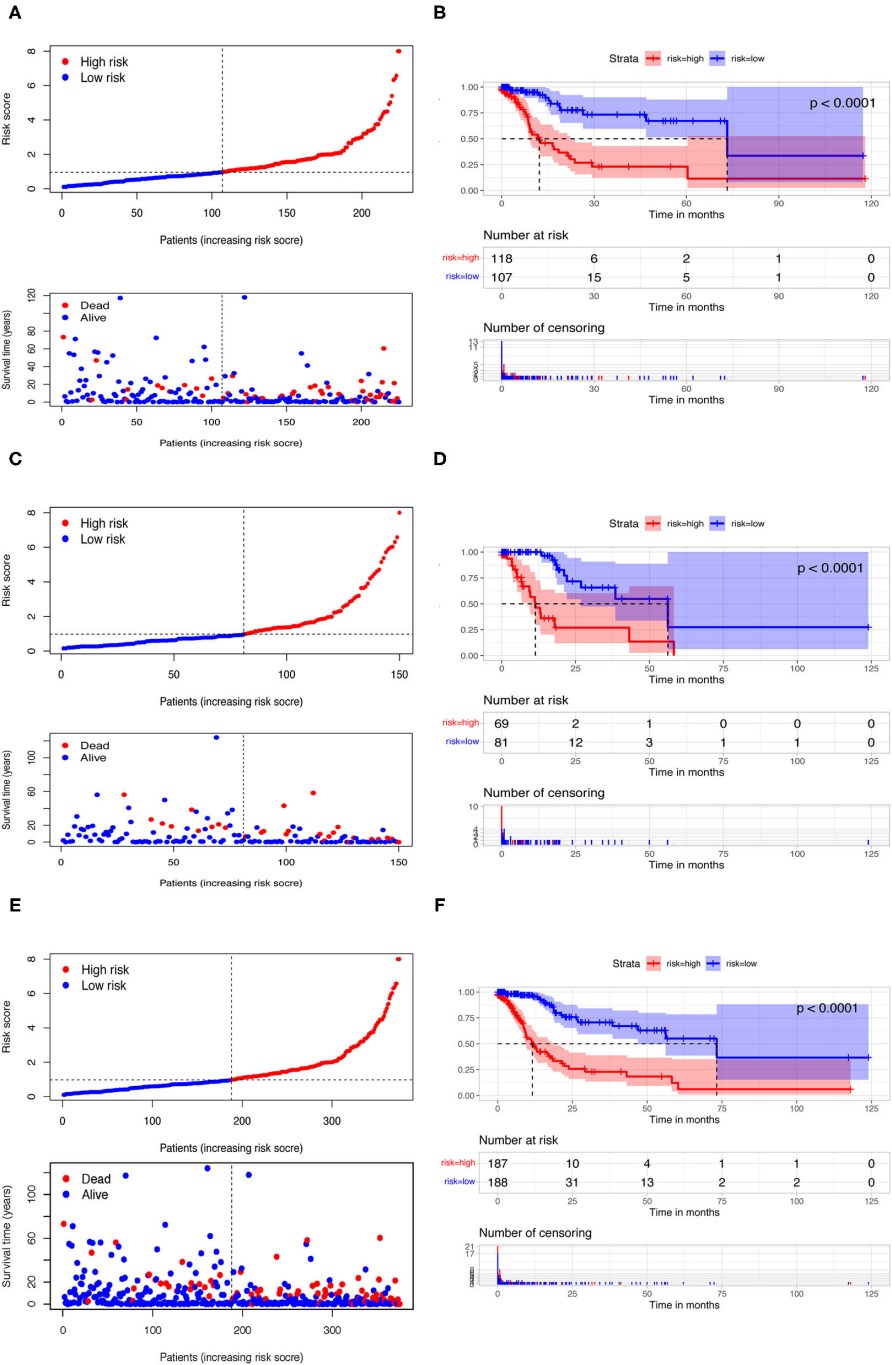
Risk assessment model for prognosis prediction. Patients in training **(A)**, testing **(C)**, and the whole cohort **(E)** were scored and divided into high-risk group (red) and low-risk group (blue). A scatter plot of patient survival was then plotted according to the size of the risk score, with blue representing living patients and red representing death; the OS in the low- and high-risk groups in three cohorts were presented by Kaplan-Meier curve analysis **(B,D,F)**.

Among 161 DEirlncRNAs, 9,326 valid DEirlncRNA pairs were determined using an iterative loop and 0–1 matrix screen. Eighty-one pairs showed a remarkable correlation with OS (*P* < 0.05). Further analysis of 81 DEirlncRNA pairs through LASSO regression analysis (Figures 2C,D) identified 31 DEirlncRNA pairs with a better correlation with OS. Finally, 10 DEirlncRNA pairs were chosen to construct the prognostic model using multivariate Cox regression analysis (Figure 2E). Next, patients in all cohorts were divided into low and high-risk groups according to median risk score (Figures 3A,C,E). Patients in the high-risk group had shorter survival times than those in the low-risk group (*p* < 0.0001) by Kaplan-Meier analysis (Figures 3B,D,F).

In order to eliminate possible model instability caused by random grouping of validation group and training group, we conducted a 10-fold cross validation, taking average AUC value to verify accuracy of the model algorithm. The average AUC is 0.7582, indicating stable reliability of our model.

### Analysis of Clinical Characteristics Through Risk Assessment Models

**Figure 4:**
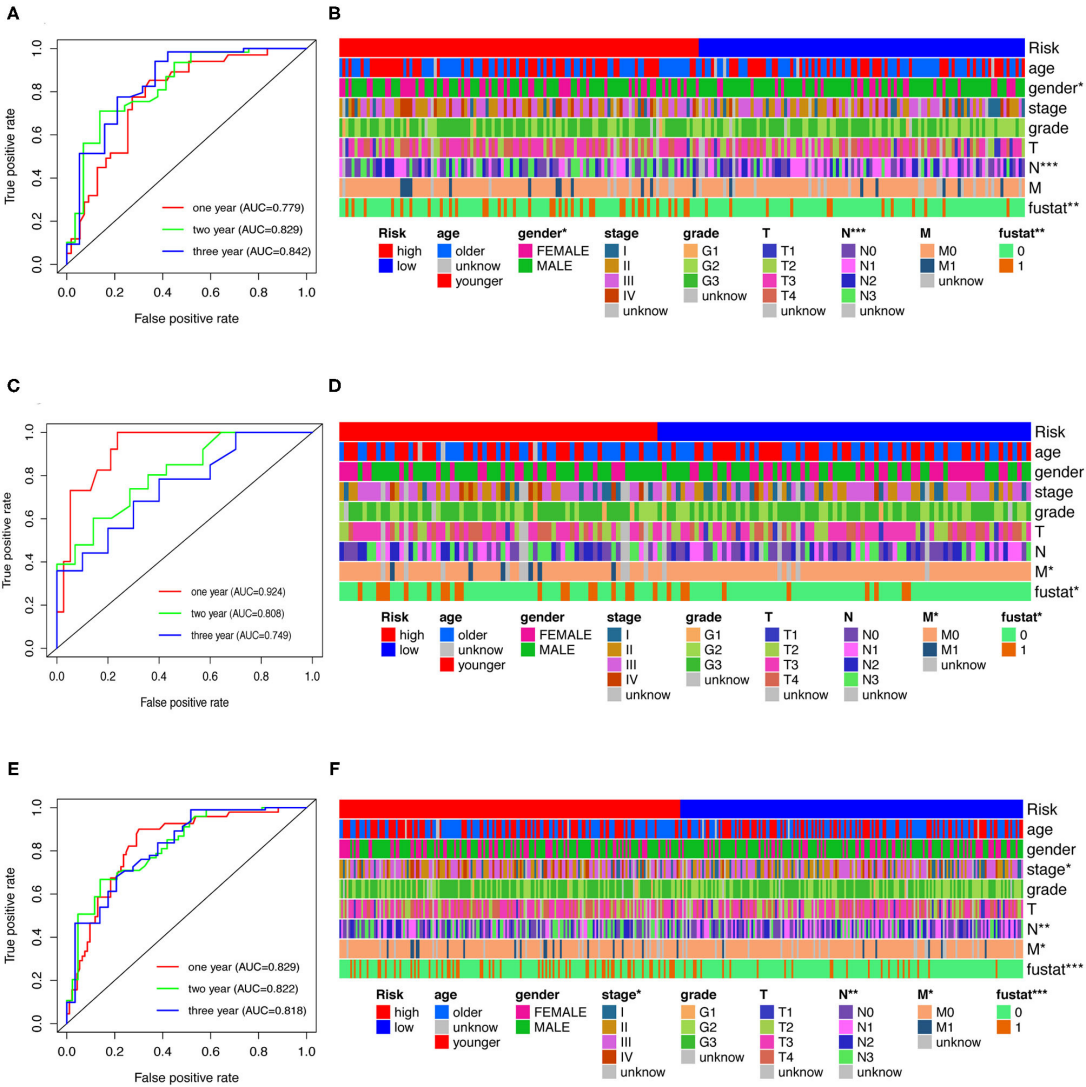
Analysis of clinical characteristics through risk assessment models. **(A,C,E)** ROC curves at 1-, 2- and 3-year post-diagnosis of the model in GC in training, testing, and the whole cohort. **(B,D,F)** A strip chart along with the scatter diagram shows that age, gender, clinical stage, tumor grade, TNM stage, and survival status were significantly associated with the risk score.

To evaluate the model, we plotted ROC curves at 1, 2, and 3 years and the model's AUCs to predict overall survival in GC patients. The AUC values were 0.779, 0.829, and 0.842, respectively, in the training cohort (Figure 4A). In the testing and whole cohort, we also found that AUC values were >0.75 (Figures 4C,E). The results showed that the model had better sensitivity and specificity when used to predict survival risk.

**Figure 5:**
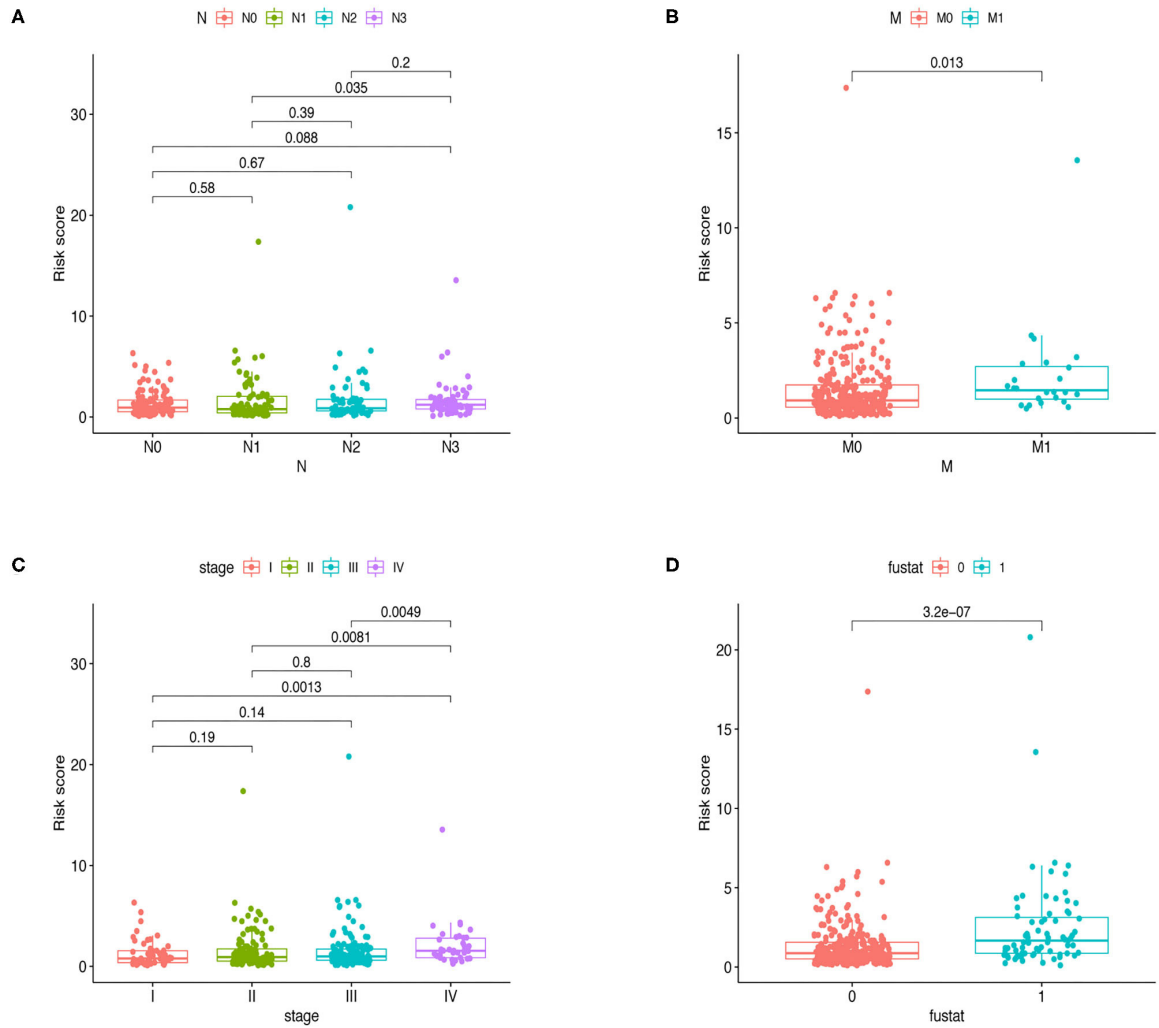
Relationships between clinical features and risk score in the whole cohort. Bar charts indicated that N stage **(A)**, M stage **(B)**, clinical stage **(C)**, and survival status **(D)** were significantly associated with risk scores in the training group.

Furthermore, Chi-square tests were used to explore the association between clinicopathological characteristics and the risk of GC (Figure 4B). The consequent scatter diagrams (Figure 5) derived from using the Wilcoxon signed-rank test indicated that N stage (Figure 5A), M stage (Figure 5B), clinical-stage (Figure 5C), and survival status (Figure 5D) were strongly correlated with a risk score. The same results were obtained in the testing and the whole cohort (Figures 4D,F).

**Figure 6:**
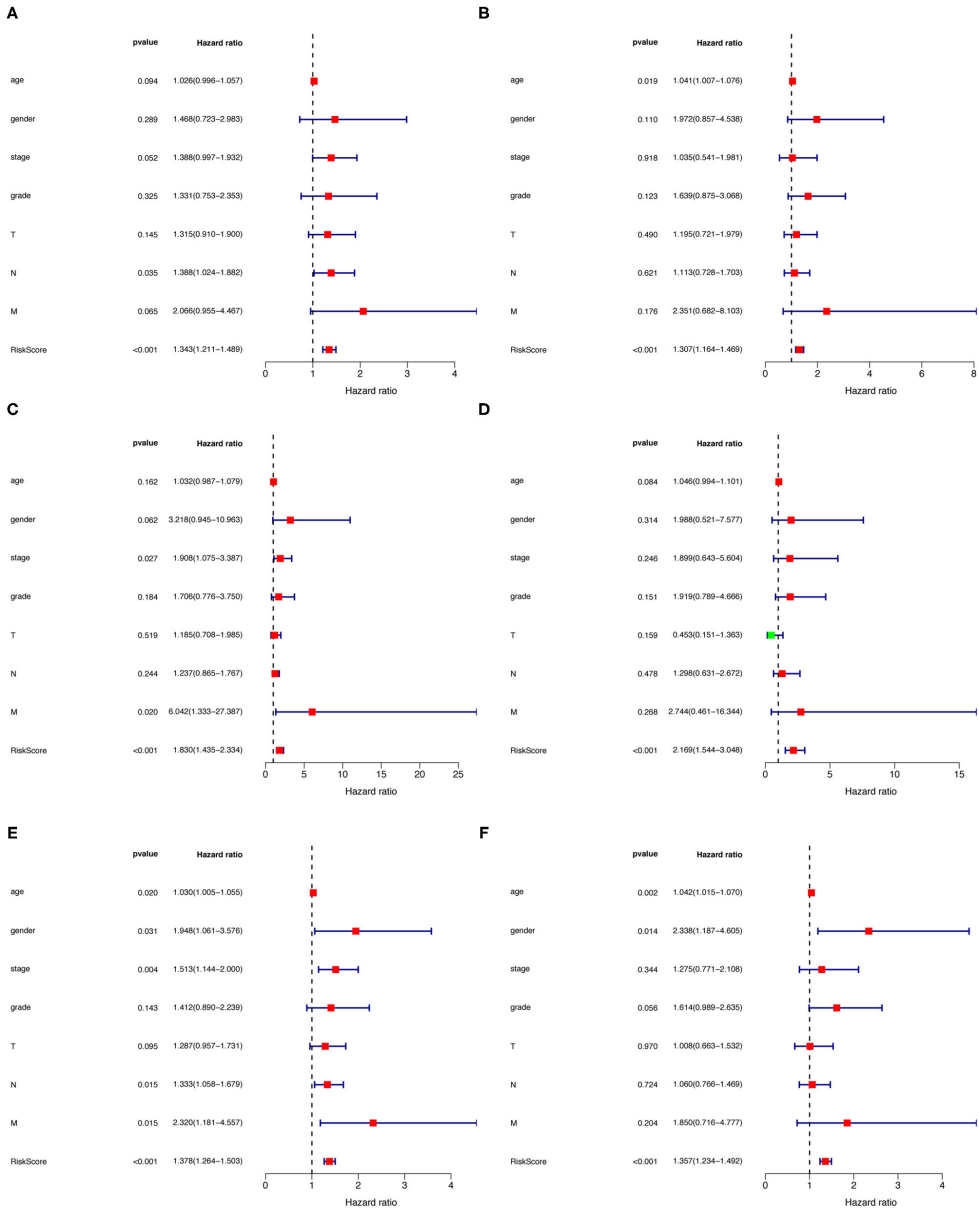
**(A–F)** Univariate and multivariate Cox regression analysis of gender, age, tumor stage, and risk score in training **(A,B)**, testing **(C,D)**, and the whole cohort **(E,F)**.

Univariate and multivariate Cox regression analyses were utilized to investigate whether the risk assessment model was an independent prognostic factor for GC in the training cohort (Figures 6A,B). We also performed nomograms based on multivariate regression analysis via the *rms* package in R software and drew line segments with the scale on the same plane according to proportion for visualization (Supplementary Figure 1). The hazard ratio (HR) of risk score and 95% confidence interval (CI) were 1.378 and 1.264–1.503 in univariate Cox regression analysis (p < 0.001), and 1.357 and 1.234–1.492 in multivariate Cox regression analysis (p < 0.001), respectively, suggesting that the risk assessment model was a prognostic factor in patients with GC. Supplementary Table 2 contains detailed values for univariate and multivariate Cox regression analyses. Among them, the risk score resulted as an independent prognostic factor in the testing cohort (Figures 6C,D) and the whole cohort (Figures 6E,F).

### Estimating Immunosuppressive Molecules and Tumor-Infiltrating Immune Cells Using Risk Assessment Models

**Figure 7:**
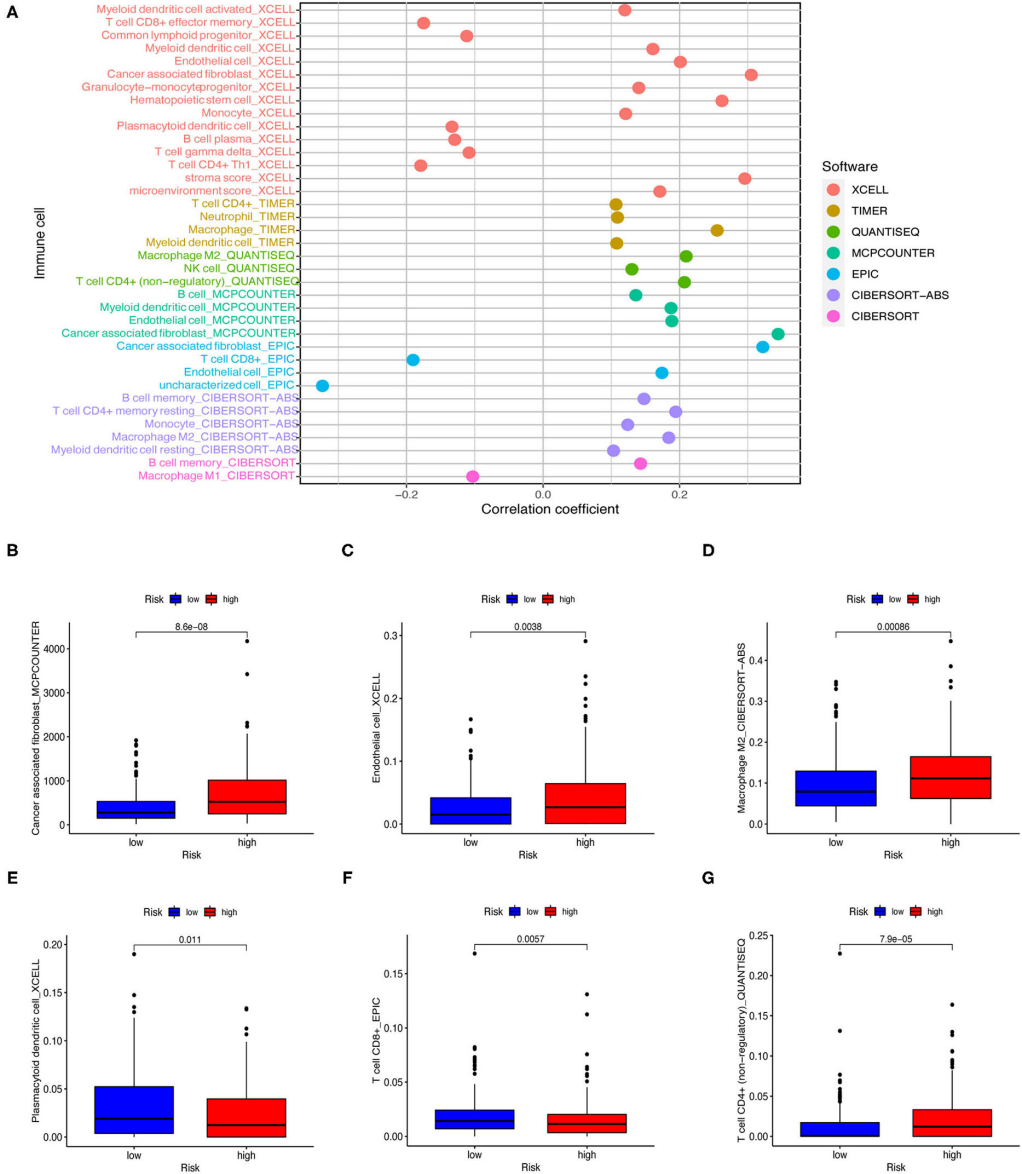
Estimating tumor-infiltrating immune cells using risk assessment models. **(A)** Patients of the training cohort were more positively associated with tumor-infiltrating immune cells in the high-risk group. **(B–G)** Patients of the testing cohort were positively associated with cancer-associated fibroblasts **(B)**, endothelial cells **(C)**, and macrophages **(D)**, and negatively associated with plasmacytoid dendritic cells **(E)**, follicular helper T cells **(F)**, and CD4+ T cells **(G)**. All figures were analyzed by Spearman correlation analysis.

As the model relies on irlncRNAs, we subsequently investigated whether the model was associated with the tumor immune microenvironment (Figure 7A). Using the Wilcoxon signed-rank test, we found that the high-risk group was positively correlated with tumor-infiltrating immune cells such as endothelial cells, macrophages, and cancer-associated fibroblasts (Figures 7B–D), and negatively correlated with CD4^+^ T cells, plasmacytoid dendritic cells, and follicular helper T cells (Figures 7E–G).

**Figure 8:**
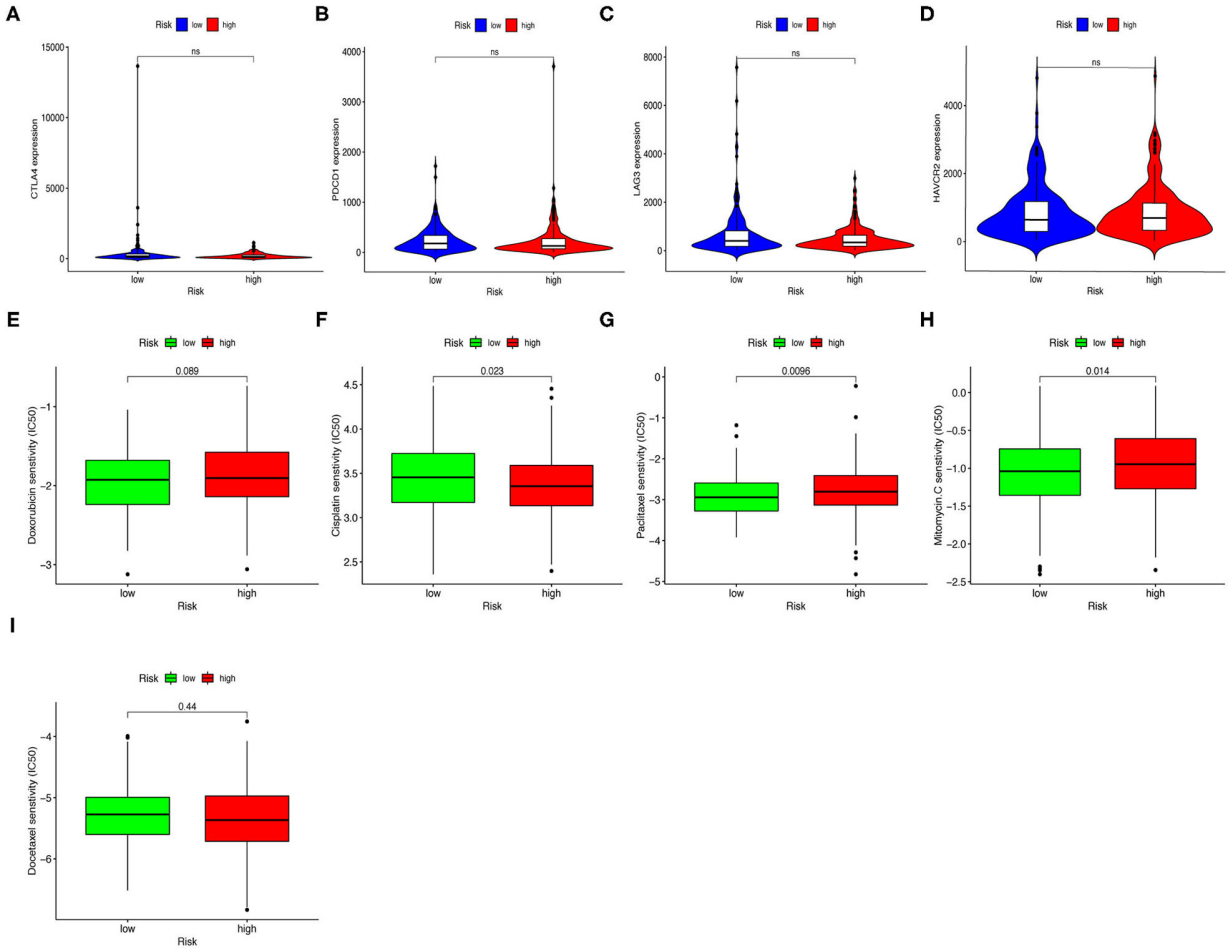
Estimating Immunosuppressed Molecules using risk assessment models. **(A–D)** High-risk scores were uncorrelated with CTLA4 **(A)**, PDCD1 **(B)**, LAG3 **(C)**, and HAVCR2 **(D)** levels; these results were not significantly different. **(E–I)** The model served as a potential predictive factor for chemotherapy sensitivity, as high-risk scores were associated with lower IC_50_ for chemotherapeutics such as cisplatin and docetaxel, and higher IC_50_ for paclitaxel, mitomycin, and doxorubicin.

Next, we investigated whether risk assessment models were associated with the expression of ICI-related biomarkers. Nevertheless, these results were not significantly different (Figures 8A–D).

### Correlation Analysis Between Risk Assessment Models and Chemotherapy Drugs

In addition to checkpoint blockade therapy, we attempted to determine the association between risk assessment models and common chemotherapeutic agents' efficacy to treat GC. We found that high-risk score was associated with lower half inhibitory concentration (IC_50_) of cisplatin (*p* = 0.023, Figure 8E) and docetaxel (*p* = 0.44, Figure 8F), and higher IC_50_ for paclitaxel (*p* = 0.0096, Figure 8G), mitomycin (*p* = 0.014, Figure 8H), and doxorubicin (*p* = 0.089, Figure 8I). Though the results of docetaxel and doxorubicin showed no significant difference in patients with GC, the above results suggested that this risk-assessed model may be a potential predictor for chemosensitivity.

### Enrichment Analysis

**Figure 9:**
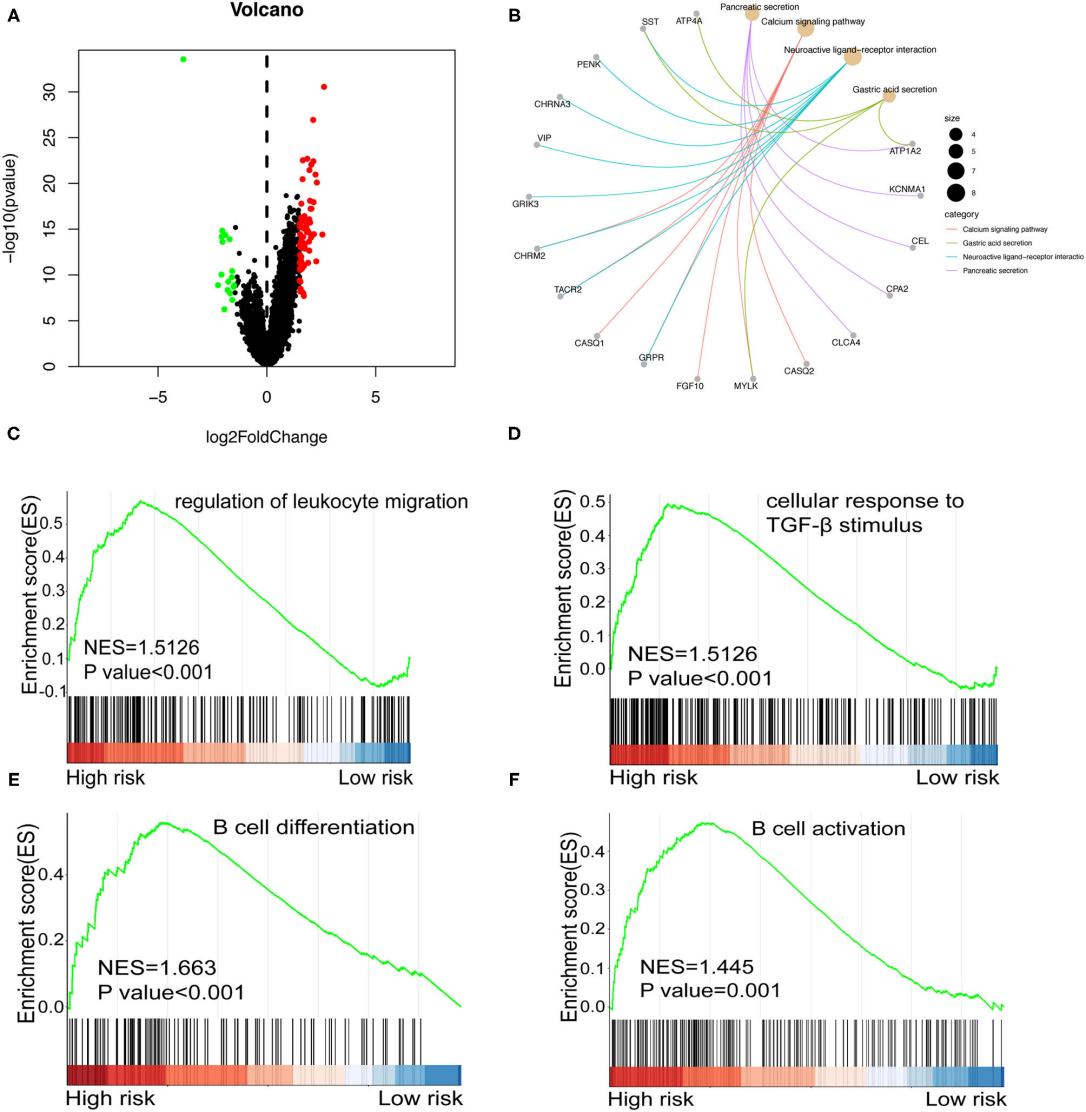
**(A)** Volcano plot shows differentially expressed genes between the high and low-risk groups in the whole cohort. Red and green dots represent up- and down-regulated irlncRNA, respectively. **(B)** KEGG Enrichment analysis indicating the biological process risk score was mainly involved in the calcium signaling pathway. **(C–F)** GSEA between low- and high-risk groups revealing regulation of leukocyte migration pathway **(C)**, cellular response to TGF-β stimulus pathway **(D)**, B cell differentiation pathway **(E)**, and B cell activation pathway **(F)**.

Subsequently, we analyzed differentially expressed genes (DEGs) in all cohort's low- and high-risk groups. Ninety DEGs (17 down-regulated genes and 73 up-regulated genes, *p* < 0.05) were (Figure 9A). Next, we conducted KEGG and GSEA enrichment analysis to further clarify biological processes related to the risk score. As shown in Figure 9B, enrichment analysis indicated that KEGG was mainly enriched in the calcium signaling pathway, gastric acid secretion, neuroactive ligand-receptor interaction, and pancreatic secretion. In the GSEA enrichment analysis, regulation of leukocyte migration pathway (Figure 9C), cellular response to transforming growth factor-β (TGF-β). TGF-β stimulus pathway (Figure 9D), B cell differentiation pathway (Figure 9E), and B cell activation pathway (Figure 9F) were notably enriched in the high-risk group.

## Discussion

Gastric cancer is one of the most common malignant tumors and a highly heterogeneous disease (2, 7). A high degree of heterogeneity is seen in the phenotype and genotype of tumor cells and the tumor microenvironment (22). GC tissue comprises both GC cells and normal cells, such as stromal cells, immune cells, and fibroblasts, which interact and co-evolve, ultimately forming a complex whole (23). Yet, the mechanisms of GC progression are still unclear; thus, identification of novel targets is urgently required.

lncRNA participates in the occurrence, development, invasion, and metastasis of GC through various ways (24–28). For example, lncRNA *MALAT1* is involved in the gene expression process and post-transcriptional regulation of mRNA splicing process (29). It also promotes tumors progression, including breast cancer, liver cancer, prostate cancer, colon cancer, and uterine cancer. Previous studies have shown that the expression level of *MALAT1* in lung cancer is higher than that in normal lung tissues, and the overall survival rate of lung cancer patients with high expression of *MALAT1* is lower (30). Also, several studies suggested that lncRNA *LUCAT1* induces a variety of malignancies related to ovarian cancer, breast cancer, renal carcinoma, and thyroid cancer. This type of lncRNA is highly expressed in many malignant tumors, including liver cancer, and is related to clinicopathological features of cancer patients (31). Thus, lncRNAs have been suggested as potential diagnostic markers and therapeutic targets of multiple cancers (32, 33).

lncRNAs are also closely related to tumor immunity. Hu *et al*. reported that long non-coding RNA *LINK-A* specifically expressed in human tissue induces metastatic breast cancer in mice by reducing phosphorylation of E3 ubiquitin ligase TRIM71 mediated by protein kinase A (34). Furthermore, Li *et al*. suggested that tumor-derived lncRNA *TUC339* is involved in the regulation of macrophage activation, and has an essential role in the regulation of macrophage M1/M2 polarization (35). In addition, Zhao and colleagues found that lncRNAs *SNHG14* / microRNA *miR-5590-3p*/ gene *ZEB1* positive feedback loop promotes progression and immune evasion of diffuse large B cell lymphoma (DLBCL) through regulating PD-1/PD-L1 checkpoint, which suggests that targeting *SNHG14* could be a promising way to improve the effectiveness of DLBCL immunotherapy (36).

Immune-related lncRNAs can be used as potential prognostic biomarkers and latent therapeutic targets. For example, overexpression of lncRNA *LINP1* restores the metastatic effect of p53, a regulator of *LINP1*. In addition, *LINP1* is up-regulated in 5-fluorouracil- and doxorubicin-resistant breast cancer cells and induces chemoresistance. *LINP1* can also inhibit breast cancer cell apoptosis induced by chemotherapeutic drugs (37). Those results indicate that *LINP1* may be a potential oncogene and chemoresistance regulator and a potential immunotherapy target in breast cancer (37). Another lncRNA, *UCA1*, has an important role in anti-tumor drug resistance. Its overexpression is related to the resistance of chemotherapeutic drugs. *UCA1* down-regulates gene *CREB1* expression by sponging microRNA *miRNA-590-3p*, promoting cells proliferation and invasion of gastric cancer, thus acting as an oncogene. Knockout of *UCA1* increases drug sensitivity of various cancers, including gastric cancer (38).

Previous studies have shown that immune-related lncRNAs and tumor immune infiltration signatures have good prognostic value in diagnosing and evaluating GC. Cao et al. constructed the immune-related lncRNAs signature and confirmed that the signature was a reliable, and independent prognostic factor that was significantly positively correlated with the infiltration of immune cells in the tumor microenvironment and the expression of key immune checkpoints (39). Moreover, Song *et al*. constructed a signature based on eight lncRNAs and found 4 key immune-related genes (*LIG1, TBX1, CTSG*, and *CXCL12*) in bladder urothelial carcinoma (40). Ma and colleagues constructed and verified a robust signature of 8 immune-related lncRNAs for the prediction of breast cancer patient survival (41). In this study, we established a model based on immune-related lncRNA and then used univariate and multivariate Cox regression and LASSO regression analysis to verify the clinical characteristics, chemotherapy drugs, and immunotherapy according to this model. The risk assessment model showed a good predictive performance and classified GC patients into high-risk and low-risk groups.

Specifically, we retrieved the original data from TCGA and carried out a co-expression analysis to identify DEirlncRNAs; we used the 0 or 1 matrix to verify lncRNA pairs. Next, 10 vital DEirlncRNAs pairs were selected using LASSO regression and univariate Cox analysis, based on which a novel assessment model was constructed. We scored the risk of the model and divided the training into low-risk and high-risk groups based on the median score. The prognostic prediction efficacy of the risk score was validated from several aspects. Firstly, ROC curves and Kaplan-Meier analysis were performed, which indicated that the risk model had better prognostic value and survival time exhibition than other factors. Secondly, in order to investigate the feasibility of prognostic markers in clinical features, we analyzed the age, gender, pathological stage, and other clinical indicators of GC patients and evaluated the association between risk score and clinical characteristics. The patients divided by risk score showed significantly different characteristics. The model was subsequently utilized to analyze tumor immune infiltration, chemotherapy efficacy in GC, and biomarkers associated with checkpoint inhibitors. Finally, enrichment analysis of the KEGG and GSEA pathways showed several notably enriched pathway signals. Patients in the high-risk group were enriched in the B cell differentiation pathway, B cell activation pathway, cellular response to transforming growth factor-beta stimulus pathway, and regulation of leukocyte migration. The literature shows that these pathways are strongly linked to the immune process; still, more evidence is needed to support this hypothesis. In addition, the research results also revealed the underlying molecular mechanism, providing a promising direction for immunotherapy.

This study has some limitations. The original dataset used for the preliminary analysis was obtained from the TCGA database, which lacks objectivity. Therefore, it was not possible to simultaneously search for data sets of other databases that support clinicopathological characteristics, lncRNA expression, and survival results of patients with GC. Yet, we employed a 0–1 matrix to select lncRNA pairs in order to reduce errors caused by expression changes. In addition, we also used single factor and multi-factor analysis, LASSO regression analysis, ROC curve, and other methods to validate the new model, which was optimized and applied. In our future study, we plan to collect clinical samples and expand the sample size for further validation.

## Conclusion

This prognostic model showed independent prognostic significance in GC. The results suggested that predicting the prognosis of GC patients without detecting lncRNA-specific expression levels could be used as a potential approach for predicting the survival of GC patients, offering a potential lncRNA target for immunotherapy.

## Data Availability Statement

The datasets presented in this study can be found in online repositories. The names of the repository/repositories and accession number(s) can be found in the article/Supplementary Material.

## Author Contributions

TB and ZW designed the study and wrote and revised the manuscript. JX reviewed the manuscript. All authors read and approved the final manuscript.

## Conflict of Interest

The authors declare that the research was conducted in the absence of any commercial or financial relationships that could be construed as a potential conflict of interest.

## Publisher's Note

All claims expressed in this article are solely those of the authors and do not necessarily represent those of their affiliated organizations, or those of the publisher, the editors and the reviewers. Any product that may be evaluated in this article, or claim that may be made by its manufacturer, is not guaranteed or endorsed by the publisher.
