# Supplementary material for: Immune-Related lncRNAs Pairs to Construct a Novel Signature for Predicting Prognosis in Gastric Cancer
Source: Front Surg. 2022 Mar 23;9:807778. doi: 10.3389/fsurg.2022.807778 (PMC8985853; doi:10.3389/fsurg.2022.807778)
Supplement: Supplementary file 3 [file Image_1.PDF]

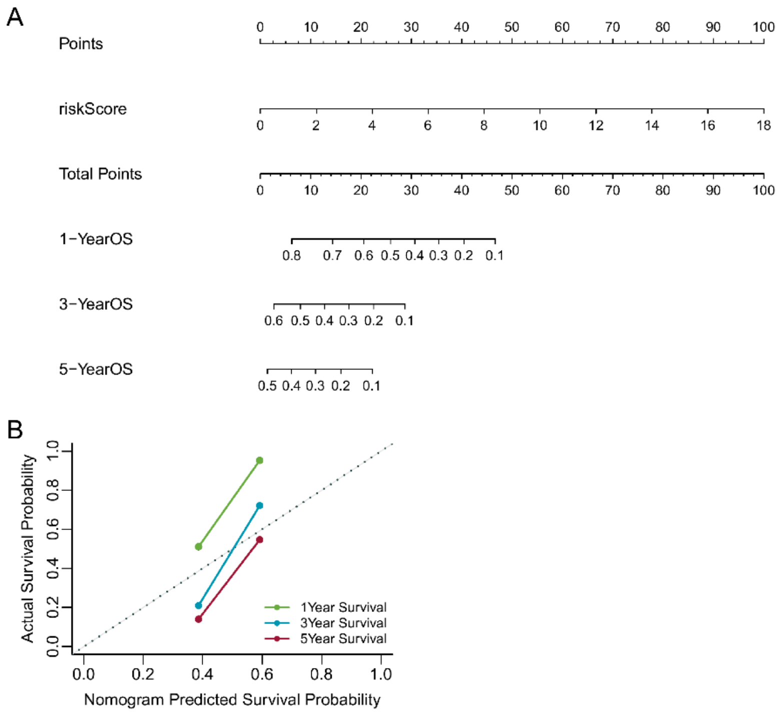

Supplemental Figure 1. Nomogram based on multivariate regression analysis via the rms package. c-index = 0.7956.
